# Descriptive Epidemiology of Diabetes Prevalence and HbA1c Distributions Based on a Self-Reported Questionnaire and a Health Checkup in the JPHC Diabetes Study

**DOI:** 10.2188/jea.JE20130196

**Published:** 2014-11-05

**Authors:** Yusuke Kabeya, Masayuki Kato, Akihiro Isogawa, Yoshihiko Takahashi, Yumi Matsushita, Atsushi Goto, Hiroyasu Iso, Manami Inoue, Tetsuya Mizoue, Shoichiro Tsugane, Takashi Kadowaki, Mitsuhiko Noda

**Affiliations:** 1 Department of Diabetes Research, National Center for Global Health and Medicine, Tokyo, Japan; 1 国立国際医療研究センター 糖尿病研究部; 2 Department of Internal Medicine, Saiseikai Central Hospital, Tokyo, Japan; 2 東京都済生会中央病院 内科; 3 Fiore Kenshin Clinic, Tokyo, Japan; 3 フィオーレ健診クリニック; 4 Department of Internal Medicine, Mitsui Memorial Hospital, Tokyo, Japan; 4 三井記念病院 内科; 5 Division of Diabetes and Metabolism, Iwate Medical University School of Medicine, Morioka, Japan; 5 岩手医科大学 内科 糖尿病・代謝内科分野; 6 Department of Clinical Research Coordination, Center for Clinical Sciences, National Center for Global Health and Medicine, Tokyo, Japan; 6 国立国際医療研究センター 臨床研究センター 臨床研究支援部; 7 Public Health, Department of Social and Environmental Medicine, Osaka University Graduate School of Medicine, Osaka, Japan; 7 大阪大学大学院医学研究科社会環境医学講座 公衆衛生学; 8 Epidemiology and Prevention Division, Research Center for Cancer Prevention and Screening, National Cancer Center, Tokyo, Japan; 8 国立がんセンター がん予防・検診センター; 9 AXA Department of Health and Human Security, Graduate School of Medicine, The University of Tokyo, Tokyo, Japan; 9 東京大学大学院医学系研究科 健康と人間の安全保障（AXA）寄付講座; 10 Department of Epidemiology and Prevention, Center for Clinical Sciences, National Center for Global Health and Medicine, Tokyo, Japan; 10 国立国際医療研究センター 疫学予防研究部; 11 Department of Diabetes and Metabolic Diseases, The University of Tokyo, Tokyo, Japan; 11 東京大学大学院医学研究科糖尿病・代謝内科

**Keywords:** diabetes mellitus, prevalence, self-report, HbA1c

## Abstract

**Background:**

The present study examined the prevalence of diabetes in Japan during the late 1990s and early 2000s using the Japan Public Health Center-based Prospective Diabetes cohort. We also investigated the distributions of HbA1c values in noncompliant diabetic participants in the cohort.

**Methods:**

A total of 28 183 registered inhabitants aged 46–75 years from 10 public health center areas were included in the initial survey. The 5-year follow-up survey included 20 129 participants. The prevalence of diabetes was estimated using both a self-reported questionnaire and laboratory measurements. Among the participants who reported the presence of diabetes on the questionnaire (self-reported diabetes), the distributions of HbA1c values were described according to their treatment status.

**Results:**

The age-standardized prevalence of diabetes in 55- to 74-year-old adults was 8.2% at the initial survey and 10.6% at the 5-year follow-up. At the initial survey, among participants with self-reported diabetes, the mean HbA1c values in the participants who had never and who had previously received diabetes treatment were 7.01% (standard deviation [SD] 1.56%) and 6.56% (SD 1.46%), respectively. Approximately 15% of the participants who had self-reported diabetes but had never received diabetes treatment had an HbA1c ≥ 8.4%.

**Conclusions:**

The prevalence of diabetes increased in the JPHC cohort between the late 1990s and early 2000s. A certain proportion of participants who were aware of their diabetes but were not currently receiving treatment had poor diabetic control. Efforts to promote continuous medical attendance for diabetes care may be necessary.

## Abstract (ja)

**【背景】:**

この研究では1990年代後半と2000年代前半の日本における糖尿病有病率を厚生労働省研究班による多目的コホート研究における糖尿病研究（JPHC Diabetes study）において算出した。また、糖尿病有病者で治療を受けていない者のHbA1c値の分布についても調査した。

**【方法】:**

全国10 保健所管内の住民28,183人（46-75歳）が初期調査の対象となった。5年後調査では20,129人が対象となった。糖尿病有病率は、コホート質問票による糖尿病の自己申告と採血データによって概計した。既知糖尿病者（質問票の自己申告により糖尿病の発症が確認されている糖尿病者）のうちで、治療状況ごとにHbA1c値の分布を調査した。

**【結果】:**

年齢調整後の糖尿病の有病率は、55-74歳の成人では初期調査で8.2％、5年後調査で10.6％であった。初期調査では、既知糖尿病者の中で、未治療者と過去に治療を受けたが現在治療をしていない者のHbA1c値の分布はそれぞれ7.01%（SD1.56%）、6.56%（SD1.46%）であった。既知糖尿病者でありながら、糖尿病を未治療である人のうち約15％にあたる人がHbA1c値8.4％以上であった。

**【結論】:**

JPHC Diabetes studyにおける糖尿病有病率は1990年後半から2000年前半にかけて上昇傾向であった。糖尿病があることを知っているが治療を行っていない者の中には血糖コントロールの状態が良くない例がある程度存在することが判明した。糖尿病治療を行っていない人を減らし治療を継続するよう推進することが重要である。

## INTRODUCTION

Diabetes mellitus is a chronic metabolic disease that imposes a considerable burden on both individual patients and healthcare systems. A dramatic increase in the number of diabetic patients has been observed in Japan during the past several decades because of the aging population and changes in dietary patterns and lifestyles.^1^^,^^2^ At present, Japan has the eighth highest number of diabetic patients in the world.^3^ According to national surveys performed by the Japanese Ministry of Health, Labour, and Welfare (MHLW) in 2002^4^ and 2007,^5^ which sampled 4000–5000 people from the general population and estimated the prevalence of diabetes, the prevalence of probable diabetes was 12.8% for males and 6.5% for females in 2002 and 15.3% for males and 7.3% for females in 2007. Thus, the estimated number of diabetic people in Japan increased from 7.4 million to 8.9 million during the 5-year period.

Regarding the prevalence of diabetes according to area, many studies have reported the prevalence in a single area, while one review reported area variations in the prevalence of diabetes.^6^ However, few studies have described the prevalence of diabetes according to area across Japan using a standardized methodology. Estimating the prevalence according to area could be important for both providing diabetes care and for assessing the quality of diabetes healthcare.

The Japan Public Health Center-based (JPHC) Prospective Diabetes study examined registered inhabitants in public health center (PHC) areas across Japan in the initial survey (1998–2000) and in the 5-year follow-up survey (2003–2005) using a standardized questionnaire and laboratory measurements. The large population-based sample and strict standardization of hemoglobin A1c (HbA1c) provided an opportunity to accurately estimate prevalence of diabetes according to area and to describe the 5-year change in the prevalence between the late 1990s and the early 2000s.

In addition, the JPHC cohort enabled us to examine the glycemic control of patients with diabetes according to treatment status. The Japanese MHLW National Nutrition Survey in 2007^5^ reported that only 50.8% of diabetic patients were currently receiving diabetes treatment, although the proportion was higher than in the previous survey in 2002.^5^ This finding suggests that poor medical attendance for diabetes treatment may still be prevalent across Japan, despite increasing awareness of the clinical importance of diabetes. To clarify the situation of glycemic control in noncompliant patients with diabetes, we additionally described the distributions of HbA1c values in noncompliant diabetic participants in the cohort.

## METHODS

Data from the JPHC Study, which was a large longitudinal cohort study investigating cancer, cardiovascular disease, and other lifestyle-related diseases in Japan, were used in the present study. The details of the study design have been described elsewhere.^7^ Briefly, the JPHC Study was initiated in 1990 for Cohort I, and subjects were added in 1993 for Cohort II. The study population consisted of all registered Japanese inhabitants in 11 PHC areas ranging in age from 40 to 59 years old in Cohort I (the Ninohe PHC area in Iwate Prefecture, Yokote PHC area in Akita Prefecture, Saku PHC area in Nagano Prefecture, Ishikawa PHC area in Okinawa Prefecture, and Katsushika PHC area in Tokyo Metropolis) and from 40 to 69 years old in Cohort II (the Kasama PHC area in Ibaraki Prefecture, Kashiwazaki PHC area in Niigata Prefecture, Tosayamada PHC area in Kochi Prefecture, Arikawa PHC area in Nagasaki Prefecture, Miyako PHC area in Okinawa Prefecture, and Suita PHC area in Osaka Prefecture). The names of the PHC areas shown here are those used at that time.

The diabetes study (the JPHC Diabetes Study) was performed in all PHC areas other than the Suita PHC area. The initial survey was performed in 1998–1999 for Cohort II and in 2000 for Cohort I. Among the registered inhabitants participating in the JPHC Study, those who received annual health checkups in each PHC-administered area were recruited; a self-reported questionnaire specific to diabetes research and measurement of HbA1c was added to their routine health checkup examinations. A 5-year follow-up survey was performed in the same way in 2003–2004 for Cohort II and in 2005 for Cohort I.

A flow chart of the study participants is shown in Figure. In the present study, 28 363 participants who responded to the questionnaire were eligible for the initial survey. We excluded 180 participants because of missing anthropometric or laboratory data. Accordingly, a total of 28 183 participants (10 268 men and 17 915 women) were therefore included in the analysis of the initial survey. Regarding the 5-year follow-up survey, 20 264 participants responded to the questionnaire. Among them, 12 215 participated in both the initial and the 5-year follow-up survey, while 8049 participated in the 5-year follow-up survey only. Of these 20 264 participants, 135 were excluded because of missing data, and a total of 20 129 participants (7639 men and 12 490 women) were included. The incidence of diabetes during the 5 years among those who participated in both the initial and the 5-year follow-up survey was reported by Noda et al.^8^ A fasting blood sample, which was defined as a sample collected ≥8 hours after the last caloric intake, was collected from 11 832 participants at the initial survey and from 7296 participants at the 5-year follow-up. If a blood sample was collected <8 hours after the last caloric intake, it was classified as a casual blood sample. This study was approved by the ethics committee of the International Medical Center of Japan, which was the former name of the National Center for Global Health and Medicine.

**Figure.**
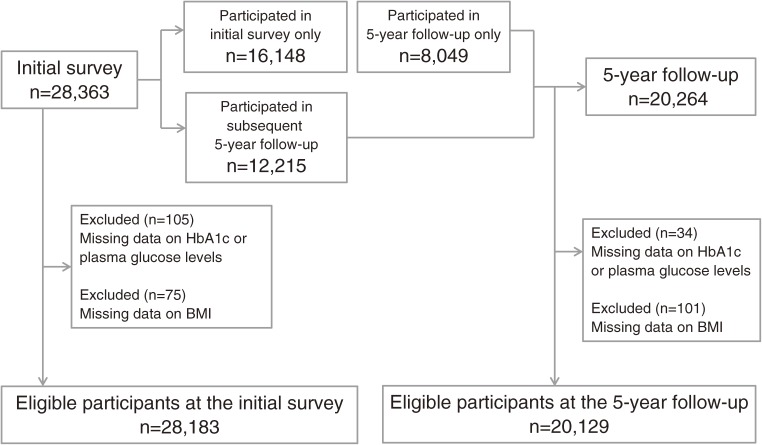
Flow chart of the study participants.

### Questionnaire used for the diabetes survey

A self-reported questionnaire regarding family history of diabetes, results of previous examinations for diabetes, physicians’ diagnosis of diabetes, current medication for diabetes, signs of diabetic complications, brief history of body weight changes, physical activity, and history of childbirth was distributed at health checkups.

### Definition of diabetes mellitus

In the present study, diabetes was defined in several ways, which are summarized in Table 1. “Self-reported diabetes” was defined as a reply to the questionnaire that met either or both of the following criteria: 1) having been told ‘you have diabetes’ by a physician, or 2) taking medication for diabetes. “Diabetes solely confirmed by laboratory data” was defined as the absence of self-reported diabetes and the presence of any of the following laboratory results: 1) a fasting plasma glucose (FPG) value of 126 mg/dL or more, 2) a casual plasma glucose value of 200 mg/dL or more, or 3) an HbA1c value of 6.5% or more in the National Glycohemoglobin Standardization Program equivalent value. “Diabetes solely confirmed by laboratory data” was referred to as “newly diagnosed diabetes” in a previously published paper regarding the prevalence of diabetes in the JPHC Study.^9^ In addition, “diabetes solely confirmed by laboratory data” was also examined using the criteria used in the clinical settings of the initial survey performed in 1998–2000. The definition was based on the World Health Organization (WHO) criteria in 1985 but was modified because results for the oral glucose tolerance test were not always available. The definition involved meeting either of the following criteria: 1) an FPG value of 140 mg/dL or more, or 2) a casual plasma glucose value of 200 mg/dL or more. To avoid confusion, “diabetes solely confirmed by laboratory data” based on the WHO criteria in 1985 was stated as “diabetes solely confirmed by laboratory data (1985 WHO)” in the present study.

**Table 1.** Definitions of diabetes used in the present study

1. Self-reported diabetes	
	• Participants who replied to the self-reported questionnaire that met either or both of the following criteria: 1) having been told ‘you have diabetes’ by a physician, or 2) taking medication for diabetes.
2. Diabetes solely confirmed by laboratory data^a^	
	• Absence of self-reported diabetes
	AND
	• Any of the following laboratory results: 1) a fasting plasma glucose (FPG) value of 126 mg/dL or more, 2) a casual plasma glucose value of 200 mg/dL or more, 3) an HbA1c value of 6.5% or more.
3. Diabetes solely confirmed by laboratory data (1985 WHO)	
	• Absence of self-reported diabetes
	AND
	• Either or both of the following laboratory results: 1) an FPG value of 140 mg/dL or more, 2) a casual plasma glucose value of 200 mg/dL or more.
4. Diabetes definition used for estimating diabetes prevalence	
	• Presence of self-reported diabetes
	OR
	• Presence of diabetes solely confirmed by laboratory data
5. Diabetes confirmed by laboratory data and/or current treatment status	
	• Any of the following criteria: 1) an FPG value of 126 mg/dL or more, 2) a casual plasma glucose value of 200 mg/dL or more, 3) an HbA1c value of 6.5% or more.
	AND/OR
	• Participants who replied to the self-reported questionnaire with “currently receiving diabetes treatment”.

Abbreviations: FPG, fasting plasma glucose; HbA1c, hemoglobin A1c; WHO, World Health Organization.

^a^“Diabetes solely confirmed by laboratory data” was referred as “newly diagnosed diabetes” in a previously published paper of the JPHC Diabetes study.^9^

Regarding the estimates of the prevalence of diabetes, diabetes was defined as the presence of “self-reported diabetes” or “diabetes solely confirmed by laboratory data.” Namely, the definition referred to any of the following criteria: 1) an FPG value of 126 mg/dL or more, 2) a casual plasma glucose value of 200 mg/dL or more, 3) an HbA1c value of 6.5% or more, or 4) self-reported diabetes. We also computed the frequency of participants (diabetes confirmed by laboratory data and/or current treatment status) who met any of the following criteria: 1) an FPG value of 126 mg/dL or more, 2) a casual plasma glucose value of 200 mg/dL or more, 3) an HbA1c value of 6.5% or more, or 4) currently receiving diabetes treatment.

### Standardization of HbA1c levels

The HbA1c measurement method differed by PHC-administered areas. Therefore, standardization of HbA1c was strictly performed to minimize variations among laboratories. Either a high-performance liquid chromatography (HPLC) assay system or an immunochemical assay system was used in each PHC, except for one PHC where the immunochemical system was changed to an HPLC system during the 5-year follow-up period. Details regarding the procedure used for standardization have been described previously.^8^ Briefly, standard samples were provided to each PHC at the time of the initial survey and the 5-year follow-up survey. The calibration procedure was conducted using the standard samples. The original standard samples were examined and approved by the Japan Diabetes Society (JDS). The procedure for HbA1c calibration used by the JDS has been described elsewhere.^9^ The averages for these standard samples were used to compute a linear regression equation using the least squares method, and the actual values were calibrated according to the regression equation. The HbA1c data were converted to equivalent values of the National Glycohemoglobin Standardization Program according to a statement made by the JDS.^10^

### Statistical analysis

The mean and standard deviation (SD) of age, body mass index (BMI), plasma glucose values, and HbA1c values were calculated according to the definitions of diabetes in the overall population at the initial survey and the 5-year follow-up. The distributions of HbA1c values were described according to the definitions of diabetes. In addition, participants with self-reported diabetes were further categorized into three groups according to their treatment status (never, previously, and currently receiving treatment), and the distributions of HbA1c values were described.

With regard to the estimation of the prevalence of diabetes, as mentioned above, diabetes was defined as the presence of “self-reported diabetes” or “diabetes solely confirmed by laboratory data.” The prevalence of diabetes was calculated at the initial survey and the 5-year follow-up in the overall population and each PHC-administered area. In order to grasp the socioeconomic characteristics of each PHC-administered area, the industrial composition was obtained from the previous JPHC report,^11^ which was based on the 1990 population census of Japan. The prevalence was standardized to the 1985 Japanese model population.^12^ The age-standardized prevalence was restricted to participants aged 55–74 years, since this was the only age range common to all PHC areas. Regarding sex-specific analysis, the prevalence standardized to a study population of each sex at the initial survey was calculated because no information on the sex-specific age distribution was included in the 1985 Japanese model population.^12^ The male and female populations at the initial survey, which were used for the standardization, were graphically confirmed to have similar age distributions. To examine time trends in the prevalence of diabetes, we used a logistic regression model fit by the generalized estimating equation method with covariate adjustment for age and sex, which took into account the participants who were included in the two surveys.^13^^,^^14^

All analyses were performed using Stata version 11 for Windows (Stata Corp., College Station, TX, USA). A value of *P* < 0.05 was considered statistically significant in the statistical tests.

## RESULTS

### Prevalence of diabetes in the JPHC diabetes cohort

The prevalences of self-reported diabetes and diabetes solely confirmed by laboratory data at the initial survey and the 5-year follow-up survey in the cohort are shown in Table 2. Of the 28 183 participants at the initial survey, 1195 participants with diabetes (4.2%) were identified by self-report and 1087 participants with diabetes (3.9%) were confirmed solely by the laboratory measurements performed at the initial survey. Thus, a total of 2282 participants had diabetes, resulting in an overall crude prevalence of 8.1%. Participants with self-reported diabetes were further categorized into three groups according to the diabetes treatment status. Of the 1195 participants with self-reported diabetes, 74.7% (893 participants) were currently receiving diabetes treatment, while the remaining participants were not currently receiving treatment (namely, they had never or had previously received treatment). If diabetes solely confirmed by laboratory data was based on the WHO criteria in 1985, the number of participants with diabetes decreased dramatically. Only 368 participants (1.3%) had diabetes solely confirmed by laboratory data according to the 1985 criteria. When diabetes was defined by laboratory data and/or current treatment status, the number of diabetic participants was 2141 (7.6%).

**Table 2.** Characteristics of study participants according to the presence of diabetes and diabetes treatment at the initial survey and 5-year follow-up

	Total		Self-reported diabetes								Diabetes confirmed solely by laboratory data^a^		Neither self- reported diabetes nor diabetes solely confirmed by laboratory data		Diabetes confirmed solely by laboratory data (1985 WHO)^b^		Neither self- reported diabetes nor diabetes solely confirmed by laboratory data (1985 WHO)^b^		Diabetes confirmed by 1) abnormal laboratory data and/or 2) currently receiving diabetes treatment^c^			

			Total		Treatment status																	
	
					Never		Previously		Currently										Yes		No	
Initial survey																						
Number of subjects	28 183		1195		161		141		893		1087		25 901		368		26 620		2141		26 042	
Age, years	62.0	(7.0)	63.9	(6.4)	63.4	(7.0)	63.1	(6.9)	64.2	(6.2)	62.8	(6.7)	61.9	(7.0)	62.2	(7.1)	62.0	(7.0)	63.4	(6.5)	61.9	(7.0)
Sex, male (%)	10 268	(36.4)	605	(50.6)	88	(55.0)	80	(56.7)	437	(48.9)	572	(52.6)	9091	(35.1)	221	(60.1)	9442	(35.5)	1105	(51.6)	9163	(35.2)
BMI	23.7	(3.2)	24.3	(3.4)	24.3	(3.4)	23.6	(2.9)	24.4	(3.4)	24.6	(3.5)	23.6	(3.1)	24.2	(3.7)	23.7	(3.2)	24.5	(3.4)	23.6	(3.1)
PG, mg/dL	106.4	(29.3)	163.4	(64.4)	156.9	(64.5)	146.4	(68.3)	167.2	(63.3)	157.2	(58.3)	101.6	(17.9)	213.4	(65.0)	102.4	(18.6)	163.4	(62.2)	101.7	(18.0)
PG (fasting)^d^, mg/dL	99.6	(19.4)	148.4	(43.3)	150.8	(50.2)	120.7	(23.4)	151.5	(42.7)	135.5	(28.2)	95.4	(9.5)	165.7	(32.3)	96.4	(10.9)	143.3	(36.8)	95.5	(9.6)
HbA1c, %	5.58	(0.70)	7.27	(1.47)	7.01	(1.56)	6.56	(1.46)	7.43	(1.41)	6.92	(1.20)	5.45	(0.39)	7.28	(1.75)	5.49	(0.46)	7.19	(1.35)	5.45	(0.40)

5-year follow-up																						
Number of subjects	20 129		1232		117		97		1018		1029		17 868		n.a.		n.a.		2151		17 978	
Age, years	66.5	(6.8)	67.7	(6.5)	67.3	(6.8)	67.9	(7.5)	67.7	(6.4)	67.2	(6.6)	66.3	(6.8)					67.4	(6.5)	66.4	(6.8)
Sex, male (%)	7639	(38.0)	613	(49.8)	56	(47.9)	66	(68.0)	491	(48.2)	506	(49.2)	6520	(36.5)					1055	(49.1)	6584	(36.6)
BMI	23.9	(3.3)	24.7	(3.5)	24.1	(3.1)	24.0	(3.2)	24.8	(3.5)	25.1	(3.9)	23.8	(3.2)					24.9	(3.7)	23.8	(3.2)
PG, mg/dL	111.0	(30.9)	162.1	(57.7)	147.5	(56.3)	147.8	(65.9)	165.1	(56.7)	154.5	(55.2)	104.9	(18.6)					160.6	(57.2)	105.0	(18.7)
PG(fasting)^e^, mg/dL	102.9	(22.1)	148.3	(44.3)	131.7	(41.7)	137.2	(49.4)	150.8	(43.7)	138.0	(28.0)	97.2	(9.3)					145.1	(37.9)	97.3	(9.4)
HbA1c, %	5.76	(0.71)	7.22	(1.23)	6.80	(1.09)	6.75	(1.69)	7.31	(1.18)	6.90	(1.04)	5.59	(0.39)					7.13	(1.16)	5.59	(0.39)

BMI, body mass index; HbA1c, hemoglobin A1c; n.a., not applicable; PG, plasma glucose; WHO, World Health Organization.

Values are the mean (SD) or *n* (%).

^a^“Diabetes solely confirmed by laboratory data” was diagnosed if a subject met any of the following criteria: 1) a fasting PG value of 126 mg/dL or more, 2) a casual PG value of 200 mg/dL or more, 3) an HbA1c value of 6.5% or more.

^b^“Diabetes solely confirmed by laboratory data (1985 WHO)” was diagnosed if a subject met either of the following criteria: 1) a fasting PG value of 140 mg/dL or more, or 2) a casual PG value of 200 mg/dL or more.

^c^Subjects who met any of the following criteria: 1) a fasting PG value of 126 mg/dL or more, 2) a casual plasma glucose value of 200 mg/dL or more, 3) an HbA1c value of 6.5% or more, 4) currently receiving diabetes treatment.

^d^A total of 11 832 participants were evaluated under fasting conditions.

^e^A total of 7296 participants were evaluated under fasting conditions.

At the 5-year follow-up survey, the crude prevalence of diabetes increased. Of the 20 129 participants, 1232 participants with diabetes (6.1%) were identified by self-reporting and 1029 (5.1%) solely by laboratory measurements. The overall crude prevalence of diabetes at the 5-year follow-up was 11.2%. Of the 1232 participants with self-reported diabetes, 82.6% (1018 participants) were currently receiving treatment. The number of participants with diabetes defined by laboratory data and/or current treatment status was 2151 (10.7%).

### Distributions of HbA1c values in different diabetic populations

Table 3 shows the distributions of the HbA1c values in different subsets of the diabetic population at the initial survey and the 5-year follow-up survey. At the initial survey, the mean (standard deviation [SD]) HbA1c values in the participants with self-reported diabetes and those with diabetes confirmed solely by laboratory data were 7.27% (SD 1.47%) and 6.92% (SD 1.20%), respectively. Among the participants with self-reported diabetes, the mean HbA1c values in the never treated, previously treated, and currently treated participants were 7.01% (SD 1.56%), 6.56% (SD 1.46%), and 7.43% (SD 1.41%), respectively. Of the participants who had self-reported diabetes but had never received diabetes treatment, 14.9% had an HbA1c ≥ 8.4% (HbA1c ≥ 8.0% for the JDS value). The corresponding proportion was 7.1% among the participants who had self-reported diabetes and had previously received diabetes treatment.

**Table 3.** Distributions of HbA1c values according to the presence of diabetes and diabetes treatment at the initial survey and 5-year follow-up

	Total		Self-reported diabetes								Diabetes confirmed solely by laboratory data^a^		Neither self-reported diabetes nor diabetes solely confirmed by laboratory data		Diabetes confirmed solely by laboratory data (1985 WHO)^b^		Neither self-reported diabetes nor diabetes solely confirmed by laboratory data (1985 WHO)^b^		Diabetes confirmed by 1) abnormal laboratory data and/or 2) currently receiving diabetes treatment^c^			

			Total		Treatment status																	
	
					Never		Previously		Currently										Yes		No	
Initial survey																						
Number of subjects	28 183		1195		161		141		893		1087		25 901		368		26 620		2141		26 042	
HbA1c, % mean (SD)	5.58	(0.70)	7.27	(1.47)	7.01	(1.56)	6.56	(1.46)	7.43	(1.41)	6.92	(1.20)	5.45	(0.39)	7.28	(1.75)	5.49	(0.46)	7.19	(1.35)	5.45	(0.40)
HbA1c, %	*n*	(%)	*n*	(%)	*n*	(%)	*n*	(%)	*n*	(%)	*n*	(%)	*n*	(%)	*n*	(%)	*n*	(%)	*n*	(%)	*n*	(%)
≥5.6	10 972	38.9	1095	91.6	141	87.6	108	76.6	846	94.7	989	91.0	8888	34.3	313	85.1	9564	35.9	1995	93.2	8977	34.5
≥6.0	3917	13.9	970	81.2	119	73.9	82	58.2	769	86.1	898	82.6	2049	7.9	271	73.6	2676	10.1	1824	85.2	2093	8.0
≥6.5	1564	5.5	791	66.2	90	55.9	54	38.3	647	72.5	773	71.1	0	0.0	224	60.9	549	2.1	1564	73.0	0	0.0
≥8.4	322	1.1	225	18.8	24	14.9	10	7.1	191	21.4	97	8.9	0	0.0	77	20.9	20	0.1	322	15.0	0	0.0
≥10.5	71	0.3	46	3.8	8	5.0	5	3.5	33	3.7	25	2.3	0	0.0	24	6.5	1	0.0	71	3.3	0	0.0

5-year follow-up																						
Number of subjects	20 129		1232		117		97		1018		1029		17 868		n.a.		n.a.		2151		17 978	
HbA1c, % mean (SD)	5.76	(0.71)	7.22	(1.23)	6.80	(1.09)	6.75	(1.69)	7.31	(1.18)	6.90	(1.04)	5.59	(0.39)					7.13	(1.16)	5.59	(0.39)
HbA1c, %	*n*	(%)	*n*	(%)	*n*	(%)	*n*	(%)	*n*	(%)	*n*	(%)	*n*	(%)					*n*	(%)	*n*	(%)
≥5.6	10 975	54.5	1182	95.9	105	89.7	85	87.6	992	97.4	979	95.1	8814	49.3					2073	96.4	8902	49.5
≥6.0	4432	22.0	1085	88.1	92	78.6	65	67.0	928	91.2	896	87.1	2451	13.7					1926	89.5	2506	13.9
≥6.5	1652	8.2	856	69.5	62	53.0	36	37.1	758	74.5	796	77.4	0	0.0					1652	76.8	0	0.0
≥8.4	250	1.2	182	14.8	15	12.8	10	10.3	157	15.4	68	6.6	0	0.0					250	11.6	0	0.0
≥10.5	48	0.2	30	2.4	1	0.9	4	4.1	25	2.5	18	1.7	0	0.0					48	2.2	0	0.0

HbA1c, hemoglobin A1c; n.a., not applicable; WHO, World Health Organization.

^a^“Diabetes solely confirmed by laboratory data” was diagnosed if a subject met any of the following criteria: 1) a fasting PG value of 126 mg/dL or more, 2) a casual PG value of 200 mg/dL or more, 3) an HbA1c value of 6.5% or more.

^b^“Diabetes solely confirmed by laboratory data (1985 WHO)” was diagnosed if a subject met either of the following criteria: 1) a fasting PG value of 140 mg/dL or more, or 2) a casual PG value of 200 mg/dL or more.

^c^Subjects who met any of the following criteria: 1) a fasting PG value of 126 mg/dL or more, 2) a casual plasma glucose value of 200 mg/dL or more, 3) an HbA1c value of 6.5% or more, 4) currently receiving diabetes treatment.

At the 5-year follow-up, the mean HbA1c values in the participants with self-reported diabetes and diabetes confirmed solely by laboratory data were 7.22% (SD 1.23%) and 6.90% (SD 1.04%), respectively. Among the participants with self-reported diabetes, the mean HbA1c values in the never treated, previously treated, and currently treated groups were 6.80% (SD 1.09%), 6.75% (SD 1.69%), and 7.31% (SD 1.18%), respectively. Regarding the patients with poorly controlled diabetes, 12.8% of the participants with self-reported diabetes who had never received diabetes treatment and 10.3% of those who had previously received treatment had an HbA1c ≥ 8.4% (HbA1c ≥ 8.0% for the JDS value).

### Prevalence of diabetes according to area

The prevalence of diabetes in each PHC-administered area across Japan is given in Table 4. In the overall population, the age-standardized prevalence, which was restricted to participants aged 55–74 years, was 8.2% at the initial survey and 10.6% at the 5-year follow-up. The difference in the prevalence of diabetes between the two surveys was statistically significant after adjustment for age and sex (*P* < 0.001). In the sex-specific analysis, the age-standardized prevalence of diabetes in men was 11.3% at the initial survey and 14.1% at the 5-year follow-up, and the age-standardized prevalence of diabetes in women was 6.5% at the initial survey and 8.6% at the 5-year follow-up. The differences in the prevalence between the two surveys were also significant in both sexes after adjustment for age (*P* < 0.001 for men; *P* < 0.001 for women).

**Table 4.** Prevalence of diabetes^a^ in PHC-administered areas across Japan

PHC-administered areas	A		B		C		D		E		F		G		H		I		J		Overall	
											
Industrial composition of the area Primary:Secondary:Tertiary (%)	25.9:32.3:41.8		19.4:29.8:50.8		26.9:37.1:36.0		11.5:30.2:58.3		0.3:40.3:59.4		10.5:41.6:47.9		25.9:44.9:29.2		26.5:25.8:47.6		37.0:19.9:43.1		28.4:24.3:47.3			
											
	Initial	5-year	Initial	5-year	Initial	5-year	Initial	5-year	Initial	5-year	Initial	5-year	Initial	5-year	Initial	5-year	Initial	5-year	Initial	5-year	Initial	5-year

Total																						
Number of subjects	3160	2345	4579	2511	4464	2571	1488	1887	734	—	5343	4677	1226	889	2018	1359	1734	385	3437	3505	28 183	20 129
Crude prevalence	8.2	11.1	7.8	9.4	8.8	9.2	9.1	12.8	6.7		8.3	13.8	5.5	10.8	8.0	9.8	6.3	5.7	9.0	11.0	8.1	11.2
Age-standardized prevalence^b^ (%)	8.2	10.3	8.1	8.5	8.7	8.2	9.2	11.8	n.a.	n.a.	8.8	13.5	5.6	11.2	8.7	8.6	6.7	5.0	8.5	11.2	8.2	10.6
Males																						
Number of subjects	1055	896	1586	919	1872	909	542	735	280	—	1876	1767	493	355	705	477	463	124	1396	1457	10 268	7639
Crude prevalence	13.0	14.6	10.5	11.5	12.5	11.2	10.3	16.6	12.5		11.7	18.3	7.1	13.2	12.2	14.0	9.5	11.3	11.7	14.2	11.5	14.6
Age-standardized prevalence^b^ (%)	13.3	13.4	10.9	11.4	12.2	9.8	9.7	15.7	n.a.	n.a.	12.4	18.7	7.9	14.4	13.1	12.2	8.7	7.0	10.6	14.8	11.3	14.1
Age-standardized prevalence^c^ (%)	13.8	14.6	11.5	11.2	13.0	10.1	10.3	16.5	n.a.	n.a.	12.4	18.7	6.7	15.1	13.7	12.6	9.0	8.4	11.7	14.4	11.7	14.4
Females																						
Number of subjects	2105	1449	2993	1592	2592	1662	946	1152	454	—	3467	2910	733	534	1313	882	1271	261	2041	2048	17 915	12 490
Crude prevalence	5.8	9.0	6.3	8.2	6.1	8.1	8.5	10.4	3.1		6.4	11.1	4.4	9.2	5.8	7.5	5.1	3.1	7.2	8.7	6.2	9.1
Age-standardized prevalence^b^ (%)	5.6	8.5	6.5	7.2	6.3	7.3	8.7	9.5	n.a.	n.a.	7.0	10.8	4.4	9.2	6.4	6.7	5.7	4.1	7.0	9.0	6.5	8.6
Age-standardized prevalence^d^ (%)	6.0	8.6	6.9	7.4	6.6	7.0	8.7	9.9	n.a.	n.a.	7.2	11.0	4.5	9.0	6.4	7.2	5.6	3.3	7.7	9.1	6.8	8.8

FPG, fasting plasma glucose; HbA1c, hemoglobin A1c; n.a., not applicable; PHC, public health center.

Age-standardized prevalence was restricted to participants aged 55–74 years because this is the only age range that is common to all areas.

^a^Diabetes was defined by any of the following criteria: 1) an FPG value of 126 mg/dL or more, 2) a casual plasma glucose value of 200 mg/dL or more, 3) an HbA1c value of 6.5% or more, 4) self-reported diabetes.

^b^Standardized to the 1985 Japanese model population.

^c^Standardized to the overall male population at the initial survey.

^d^Standardized to the overall female population at the initial survey.

As for the area-specific prevalence of diabetes, the prevalence varied widely across PHC-administered areas, ranging from 5.6% to 9.2% at the initial survey and from 5.0% to 13.5% at the 5-year follow-up. Generally, higher values were observed in the prevalence of diabetes at the 5-year follow-up survey than at the initial survey in most areas.

## DISCUSSION

The present study estimated the prevalence of diabetes in the JPHC cohort in the late 1990s and early 2000s. The large population-based sample and strict standardization of HbA1c enabled us to estimate the prevalence of diabetes with accuracy. The main finding is that the age-standardized prevalence of diabetes in 55- to 74-year-old adults was 8.2% at the initial survey and 10.6% at the 5-year follow-up, suggesting that the prevalence increased during that period. As for area variations, the present study reported a two-fold difference in the prevalence of diabetes among some regions. A similar degree of area variations has been reported in a previous study, which showed that a relatively urban area had an approximately two-fold higher prevalence of diabetes than a rural area.^15^ When looking at the industrial composition of each PHC-administered area (Table 4), wide variations were observed, which could reflect differences in local lifestyles. While it appears that two-fold area variations were observed across areas with different lifestyles in Japan, there is too little information to assess the link between urbanization and the prevalence of diabetes in the present study. Of course, there is a possibility that sampling errors could have affected the results.

Regarding the sex-specific analysis (Table 4), our data suggests that the prevalence was higher and area variations wider in men than in women. Further understanding of the differences between sexes is important for the development of targeted health promotion programs to prevent diabetes.

When the prevalence of diabetes was compared with the estimates from one review^1^ that investigated the prevalence and prevalence trends in Japan through studying published reports from 1964 to 1992, consistency of our estimates with the review’s estimates was confirmed. The prevalence of diabetes in the early 1990s was 7%–10% in men and 3%–6% in women and increasing.^1^ The estimates in the present study were on the projected regression curve shown in the review.^1^

We also compared the prevalence in the present study with the published report^4^ of the prevalence of diabetes in the national surveys performed at nearly the same period, finding that the estimates were slightly different. The prevalence of diabetes in the national survey and the present study according to age categories is shown in eTable. Although a broader definition of diabetes was adopted in the present study than in the national survey, the prevalence of diabetes was generally lower in the national survey. In addition, the prevalence in women showed a downward trend during the 5 years in the national survey, which was not observed in the present study. The discrepancy in the results should be further investigated.

Of note, the study population of the JPHC Diabetes Study consisted of participants who responded to the questionnaire. These participants might have been more health-conscious than the participants of the national survey, which might have resulted in the lower prevalence of diabetes observed in the participants of the JPHC Diabetes Study. The difference in the sampling methods between the national survey and the JPHC Diabetes Study might have also affected the discrepancy. The national survey was performed in areas selected by geographical cluster sampling across Japan, whereas the surveys of the JPHC Diabetes Study were performed in specific PHC areas. As such, there could be a possibility that low-prevalence areas might be included in the JPHC Diabetes Study. However, the large sample size and strict standardization of HbA1c strengthen the results of our survey. In the present study, increases in BMI were also observed in most age categories and both sexes, which were more prominent in males than in females. This suggests that obesity-related lifestyles could contribute to the increase in the prevalence of diabetes.

The present study identified patients with diabetes based on both a self-reported questionnaire and laboratory measurements, enabling an analysis of unrecognized diabetes and missed opportunities to access medical treatment. At the initial survey, 4.2% of the study participants were aware of their diabetes. On the other hand, almost an equivalent number of participants (3.9%) had diabetes but were unaware of it. These participants were newly diagnosed as having diabetes based on the laboratory measurements performed in the survey. Diabetes is often asymptomatic and difficult to recognize. Considering that a non-negligible number of people who are not aware of their diabetes exist in the general population, population-based screening tests for the detection of diabetes should be promoted. When the 1985 WHO criteria, which were used in the clinical setting at the time of the initial survey, were adopted to diagnose diabetes, the number of participants with diabetes solely confirmed by laboratory data (1985 WHO) was relatively small. This result suggests that the self-reported questionnaire could provide a valid estimate of the prevalence of diabetes defined according to the 1985 WHO criteria.

In the present study, high proportions of medical attendance were reported among participants with self-reported diabetes. The proportion of those who were currently receiving diabetes treatment was 75% at the initial survey and increased to 83% at the 5-year follow-up. An increase in the proportion of participants who were currently receiving diabetes treatment was also reported in a national survey,^5^ which is consistent with the findings of the present study. The upward trend and the high proportions of patients receiving treatment could reflect increased public awareness of diabetes. Once people recognize their diabetes, they are likely to access healthcare and receive medical treatment.

The distributions of HbA1c values among participants who had never received, previously received, or were currently receiving treatment for diabetes showed that the mean HbA1c value was highest among those who were currently receiving diabetes treatment. This result seems reasonable, since these patients might include those who had a poor response to diabetes treatment or those with more advanced disease. The mean HbA1c values in the participants who had never or previously received diabetes treatment were unexpectedly fair. However, it should be considered that the study population consisted of participants attending health checkups, and these participants might have therefore been more health conscious than those who did not participate. The glycemic control in the noncompliant groups may be worse if the survey were applied to the whole population, including those who do not participate in health checkups.

Also of note, poor diabetic control was observed among participants who had never or previously received diabetes treatment. At the initial survey, 14.9% of the participants who were aware of the presence of diabetes but had never received diabetes treatment had an HbA1c ≥ 8.4%. This finding implies that a certain proportion of diabetic participants were left untreated despite their awareness of diabetes. An effort to encourage continuous medical attendance should be promoted to reduce the number of untreated diabetes.

The present study had some limitations. First, the JPHC cohort consists of health checkup participants. Thus, whether the results can be applied to the whole population is uncertain. Another limitation was the differences in the study participants between the initial survey and the 5-year follow-up survey. Of the participants in the initial survey, those who did not participate in the 5-year follow-up survey had higher HbA1c levels (data not shown) and were more likely to have diabetes at the initial survey than those who did. This follow-up bias could have affected the results. As for the type of diabetes, the present study did not distinguish between type 1 and type 2 diabetes. Since the prevalence of type 1 diabetes is very low in Japan, the vast majority of the participants with diabetes were thought to have type 2 diabetes. In fact, of the 2282 participants with diabetes at the initial survey, only 107 participants (4.7%) were on insulin treatment, which was confirmed by the self-reported questionnaire. The validity of self-reported diabetes is another concern in the present study. A self-reported questionnaire always involves misclassification. However, one past study^16^ demonstrated high specificity of self-reported diabetes in a similar setting in Japan. This suggests that, although a self-reported questionnaire is not perfect, participants with self-reported diabetes were likely to have true diabetes.

In summary, the present study assessed the growing burden of diabetes and estimated prevalence of diabetes among participants across Japan in the late 1990s and early 2000s. The 5-year change in the prevalence of diabetes in the JPHC Study was increasing, and wide variations in the prevalence were observed across the different study areas. A concerted effort to reduce the number of individuals with unrecognized or untreated diabetes is required to stop the diabetes epidemic.

## ONLINE ONLY MATERIALS

eTable. Comparison of the prevalence of diabetes between the national surveys and the present study.

Abstract in Japanese.
