# Supplementary material for: Descriptive Epidemiology of Diabetes Prevalence and HbA1c Distributions Based on a Self-Reported Questionnaire and a Health Checkup in the JPHC Diabetes Study
Source: J Epidemiol. 2014 Nov 5;24(6):460–8. doi: 10.2188/jea.JE20130196 (PMC4213220; doi:10.2188/jea.JE20130196)
Supplement: eTable. [file je-24-460-s001.pdf]

eTable. Comparison of the prevalence of diabetes between the national surveys and the present study

Survey	National Diabetes survey		JPHC Diabetes study		JPHC Diabetes study	
Year	1997	2002	1998-2000	2003-2005	1998-2000	2003-2005
Definition of diabetes	1) HbA1c ≥ 6.5% or 2) currently being treated for diabetes		1) FPG ≥ 126 mg/dl; 2) Casual PG ≥ 200 mg/dL; 3) HbA1c ≥ 6.5%; or 4) Self-reported diabetes			
Men						
Number of	1,852	1,748	10,268	7,639	10,268	7,639
	Prevalence in 10-year age categories (%)		Prevalence in 5-year age categories (%)		BMI in 5-year age categories (kg/m^2)	
Age category, years						
40-44	5.4	4.4				
45-49			7.1		24.0	
50-54	14.2	14.0	10.8	11.1	23.9	24.4
55-59			9.7	13.0	23.8	24.1
60-64	17.5	17.9	11.7	13.5	23.8	24.3
65-69			12.6	14.9	23.5	24.0
70-74			11.8	15.9	22.9	23.8
75-79	11.3	21.3	14.7	15.5	22.4	23.0
80-84				15.6		22.8
Women						
Number of	2,680	2,487	17,915	12,490	17,915	12,490
	Prevalence in 10-year age categories (%)		Prevalence in 5-year age categories (%)		BMI in 5-year age categories (kg/m^2)	
Age category, years						
40-44	5.3	3.6				
45-49			2.7		23.3	
50-54	7.1	4.6	3.7	5.6	23.3	23.5
55-59			5.0	7.7	23.7	23.5
60-64	10.5	11.5	6.3	7.9	23.9	24.0
65-69			7.9	9.3	24.0	24.1
70-74			7.9	10.7	23.7	24.1
75-79	15.5	11.6	6.8	11.6	23.5	23.8
80-84				12.5		23.2

BMI, body mass index; FPG, fasting plasma glucose; HbA1c, hemoglobin A1c; JPHC, Japan Public Health Center.
