# Supplementary material for: Descriptive Epidemiology of Diabetes Prevalence and HbA1c Distributions Based on a Self-Reported Questionnaire and a Health Checkup in the JPHC Diabetes Study
Source: J Epidemiol. 2014 Nov 5;24(6):460–8. doi: 10.2188/jea.JE20130196 (PMC4213220; doi:10.2188/jea.JE20130196)
Supplement: Abstract in Japanese. [file je-24-460-s002.pdf]

http://dx.doi.org/10.2188/jea.JE20130196

糖尿病有病率と HbA1c の分布：厚生労働省研究班による多目的コホート研究における糖尿病研究（JPHC Diabetes study）の調査結果から

壁谷悠介 1,2、加藤昌之 1,3、五十川陽洋 4、高橋義彦 5、松下由実 6、後藤温 1、磯博康 7、井上真奈美 8,9、溝上哲也 10、津金昌一郎 8、門脇孝 11、野田光彦 1

1 国立国際医療研究センター　糖尿病研究部

2 東京都済生会中央病院　内科

3 フィオーレ健診クリニック

4 三井記念病院　内科

5 岩手医科大学　内科　糖尿病・代謝内科分野

6 国立国際医療研究センター　臨床研究センター　臨床研究支援部

7 大阪大学大学院医学研究科社会環境医学講座　公衆衛生学

8 国立がんセンター　がん予防・検診センター

9 東京大学大学院医学系研究科 健康と人間の安全保障（AXA）寄付講座

10 国立国際医療研究センター　疫学予防研究部

11 東京大学大学院医学研究科糖尿病・代謝内科

【背景】この研究では 1990 年代後半と 2000 年代前半の日本における糖尿病有病率を厚生労働省研究班による多目的コホート研究における糖尿病研究（JPHC Diabetes study）において算出した。また、糖尿病有病者で治療を受けていない者の HbA1c 値の分布についても調査した。

【方法】全国 10 保健所管内の住民 28,183 人（46-75 歳）が初期調査の対象となった。5 年後調査では 20,129 人が対象となった。糖尿病有病率は、コホート質問票による糖尿病の自己申告と採血データによって概計した。既知糖尿病者（質問票の自己申告により糖尿病の発症が確認されている糖尿病者）のうちで、治療状況ごとに HbA1c 値の分布を調査した。

【結果】年齢調整後の糖尿病の有病率は、55-74 歳の成人では初期調査で 8.2％、5 年後調査で 10.6％であった。初期調査では、既知糖尿病者の中で、未治療者と過去に治療を受けたが現在治療をしていない者の HbA1c 値の分布はそれぞれ 7.01%（SD1.56%）、6.56%（SD1.46%）であった。既知糖尿病者でありながら、糖尿病を未治療である人のうち約 15％にあたる人が HbA1c 値 8.4％以上であった。

【結論】JPHC Diabetes study における糖尿病有病率は 1990 年後半から 2000 年前半にかけて上昇傾向であった。糖尿病があることを知っているが治療を行っていない者の中には血糖コントロールの状態が良くない例がある程度存在することが判明した。糖尿病治療を行っていない人を減らし治療を継続するよう推進することが重要である。

キーワード：糖尿病、有病率、自己申告、HbA1c
